# Light competition drives species replacement during secondary tropical forest succession

**DOI:** 10.1007/s00442-024-05551-w

**Published:** 2024-05-10

**Authors:** Tomonari Matsuo, Miguel Martínez-Ramos, Yusuke Onoda, Frans Bongers, Madelon Lohbeck, Lourens Poorter

**Affiliations:** 1 Forest Ecology and Forest Management Group, Wageningen University, P.O. Box 47, 6700 AA Wageningen, The Netherlands; 2 Instituto de Investigaciones en Ecosistemas y Sustentabilidad, Universidad Nacional Autónoma de México, CP 58190 Morelia, Michoacán México; 3 Graduate School of Agriculture, Kyoto University, Kyoto, 606-8502 Japan

**Keywords:** Functional traits, Light competition, Light interception and use, Species replacement, Tropical forest succession

## Abstract

Light competition is thought to drive successional shifts in species dominance in closed vegetations, but few studies have assessed this for species-rich and vertically structured tropical forests. We analyzed how light competition drives species replacement during succession, and how cross-species variation in light competition strategies is determined by underlying species traits. To do so, we used chronosequence approach in which we compared 14 Mexican tropical secondary rainforest stands that differ in age (8–32 year-old). For each tree, height and stem diameter were monitored for 2 years to calculate relative biomass growth rate (RGR, the aboveground biomass gain per unit aboveground tree biomass per year). For each stand, 3D light profiles were measured to estimate individuals’ light interception to calculate light interception efficiency (LIE, intercepted light per unit biomass per year) and light use efficiency (LUE, biomass growth per intercepted light). Throughout succession, species with higher RGR attained higher changes in species dominance and thus increased their dominance over time. Both light competition strategies (LIE and LUE) increased RGR. In early succession, a high LIE and its associated traits (large crown leaf mass and low wood density) are more important for RGR. During succession, forest structure builds up, leading to lower understory light levels. In later succession, a high LUE and its associated traits (high wood density and leaf mass per area) become more important for RGR. Therefore, successional changes in relative importance of light competition strategies drive shifts in species dominance during tropical rainforest succession.

**Supplementary Information:**

The online version contains supplementary material available at 10.1007/s00442-024-05551-w.

## Introduction

During tropical secondary forest succession, different plant species attain their maximum biomass at different moments in time, and hence there is a gradual species replacement (Bryan 1996; Peña-Claros 2003). In tropical rainforest, species replacement is driven by light competition as light is the most limiting resource in vertically developed tropical forests (Fauset et al. 2017; Rozendaal et al. 2020). During tropical forest succession, there is a rapid build-up of the forest canopy, resulting in a marked vertical light gradient with less light in the forest understory (i) Investing in height or crown growth to increase light interception (LIE, light interception per unit aboveground biomass) (Hikosaka et al. 1999; Falster and Westoby 2003), and/or (ii) Utilizing the intercepted light more efficiently for their growth (i.e., light use efficiency, LUE) (Valladares and Niinemets 2008; Onoda et al. 2014). Tree species differ in their light competition strategies by having different whole-tree, stem, and leaf trait values (Falster et al. 2017; Maharjan et al. 2021). However, few studies have actually quantified light competition strategies of tree species and linked them to underlying functional traits in the field (but see, Selaya et al. 2007; Van Kuijk et al. 2008), which is fundamental to understand the mechanism of species replacement during secondary tropical rainforest succession (Poorter et al. 2023).

During secondary succession, species replacement is determined by the demographic processes of the species, such as recruitment, growth, and mortality rates (Martínez-Ramos et al. 2021). Community assembly and species composition are initially determined by seed dispersal and recruitment and, hence, by the surrounding forest landscape that provides seeds and animal dispersers (Arroyo-Rodríguez et al. 2017). Previous land-use and associated soil properties can also act as filters that determine initial species composition (Jakovac et al. 2016). As succession proceeds, species dominance (i.e., the relative biomass of a species in a community) is more determined by the resource competition and biotic filters and thus by the growth rate of the species (Lohbeck et al. 2014). Hence, species’ growth rate becomes the strongest driver of species ‘replacement’ (i.e., changes in species dominance over time) (Muscarella et al. 2017). To understand to what extent inherent growth rate of species determines changes in species dominance, and how this pattern changes during succession, we use the relative biomass growth rate (RGR, Hendrik and Remkes 1990). The RGR is the aboveground biomass gain per unit aboveground biomass per year (g g^−1^ year^−1^) which allows to compare the growth rate of species and individuals that differ in size (i.e., biomass).

Light competition shapes the growth rate of tree species because of the large vertical light gradient in the forests, where tall trees capture a disproportionate amount of light and grow faster than small trees (Selaya et al. 2007; Onoda et al. 2014). To understand how light competition strategies determine growth rate of species, we broke down RGR mathematically into two underlying components: light interception efficiency which indicates the amount of intercepted light per unit aboveground biomass (LIE, in MJ g^−1^ year^−1^) and light use efficiency which indicates the amount of biomass growth per unit intercepted light (LUE, in g MJ^−1^) (see Eq. 6 in methods). Early in succession, there is a fast light attenuation rate in a canopy because of a dense stand foliage. Hence, species with high LIE may benefit more from this steeper light gradient because a small biomass investment in height growth leads to a disproportional increase in light availability (Selaya et al. 2007; Matsuo et al. 2021). In contrast, later in succession, less light is transmitted below canopy layer, and therefore high efficiency of light use for growth may become more important (Niinemets and Valladares 2016). Because most changes in structural and light attributes occur in the first 20 years of tropical forest succession (van Breugel et al. 2006; Matsuo et al. 2021), successional changes in light competition strategy and corresponding light-competition-driven species replacement should also occur in the first few decades of succession. Although the importance of light competition for successional species replacement is often inferred based on the observations, it has been hardly quantified and measured in the field.

Functional traits are morphological or physiological traits that shape the ecological strategies of species and their performance in a given environment (McGill et al. 2006; Rubio et al. 2021).Thus, they allow us to understand how tree species with different trait values deal with light competition (Van Kuijk et al. 2008; Kunstler et al. 2016). For instance, LIE is likely to increase with tree size as species with taller height can have better access to light while species with larger crown can intercept more light for a given light condition (Poorter et al. 2005; Selaya et al. 2007). LIE should also be higher for species with low wood density (WD) as they need lower biomass for a given height growth and thus can attain efficient vertical height extension and efficient light interception (Sterck et al. 2006b; Anten and Schieving 2010; Iida et al. 2012). In contrast, leaf traits such as leaf area (LA) and leaf mass per area (LMA) might be weakly related to LIE because light interception is more determined by whole canopy structure than by single leaf characteristics (Rubio et al. 2021). LUE is likely to increase with net carbon gain (i.e., a high photosynthetic capacity) in well-lit conditions and with an efficient use of resources such as a low respiration rate related to the shade tolerance in shaded conditions (Lawton 1984; Poorter 1994; Wright et al. 2004). Although functional traits underly light interception and use strategies, few studies have linked traits with quantitative measures of light competition during succession (but see, Lasky et al. 2014). Here we link ‘soft traits’ (easily measurable traits) with LIE and LUE to understand species’ light competition strategies, and hence their success during succession.

This study aims to evaluate to what extent changes in species dominance during succession can be explained by the differences in the ability of tree species to compete for light (Bartoń 2019). We do so by assessing how and to what extent species RGR determines the changes in species dominance over time, how RGR is driven by two different light competition strategies (LIE and LUE), and how these, in turn, are determined by underlying whole-tree, stem, and leaf traits. We addressed the following three questions and corresponding hypotheses: first, how does RGR predicts successional changes in species dominance? We hypothesize that RGR is positively associated with changes in species dominance throughout succession since higher RGR indicates a larger relative biomass increment per unit initial biomass. Second, how is species RGR determined by light competition strategies? We hypothesize that in short-statured early-successional forests, species RGR is mostly determined by LIE, whereas in tall and shaded later-successional forest, RGR is mostly determined by LUE. Third, how do species traits determine light competition strategies during succession? We hypothesized that species with taller height and larger crowns could increase light interception and thus LIE throughout succession. Species with lower WD might have higher LIE compared to species with higher WD because they need lower biomass for a given height growth and thus attain efficient vertical extension and light interception. For LUE, we hypothesized that in early succession, species with higher leaf nitrogen concentration have higher LUE because of a high photosynthetic capacity under well-lit conditions. In later succession, species with higher LMA and WD may have higher LUE because shade-tolerant species often have more efficient conversion of intercepted light to growth in the shaded conditions.

## Materials and methods

### Study site

Research was conducted near Loma Bonita town (16°04′ N; 90°55′W), southeast Mexico. The climate is warm and humid with a mean annual temperature of 24 °C, and mean annual precipitation of ca. 3000 mm (Martínez-Ramos et al. 2009). The vegetation consists mainly of lowland tropical rainforests and semi-deciduous forests (Ibarra-Manríquez and Martínez-Ramos 2002).

Secondary forest plots were selected on abandoned maize fields (“milpas”) in areas with undulating hills, between 115 and 300 m asl., with a complex acidic soil (pH 4–5) derived from sedimentary rocks (sandy and clay) (Siebe et al. 1995; van Breugel et al. 2007). Maize fields were established after clear cutting the original old-growth forest, used for corn cultivation once, and subsequently abandoned. All plots were bordering remnants of old-growth forest or connected to them by another secondary forest plot, and hence have similar geomorphology and land-use history. Fallow age and land-use history were determined based on information of landowners and other local residents.

### Field survey

To analyze how species replacement is driven by light competition during succession, we used chronosequence approach in which we compared 14 secondary forest stands that differed in age in 2018 (8–32 years, van Breugel et al. 2006) since agricultural abandonment. Most studied species are evergreen (57 out of 77 species), and 75 out of 77 species are classified into 3 successional guilds based on the previous studies and field observations; 23 species are classified into early-successional species, 35 into mid-successional species and 17 into late-successional species (M. Martínez-Ramos, unpublished, see more details in Table S1). The earliest successional forest in our study (8-year-old forest) was dominated by early-successional species such as *Conostegia xalapensis* and *Vismia camparaguey*, while the latest successional forests (32-year-old forests) were dominated by early and mid-successional species such as *Luehea speciosa* and *Vochysia guatemalensis* (Tables S1, S2). This indicates that latest successional forest stand in our study is still in a mid-successional stage in terms of species composition*.* We used a plot of 40 m × 10 m, and divided it into 16 subplots of 5 m × 5 m. In 2016 and 2018, all individuals thicker than 1 cm stem diameter at breast height (DBH) were mapped and identified to species, and their DBH and height were measured. Height was measured with a telescope rod or a range finder. In February 2019, for each individual, the height of the crown base (i.e., the distance between the ground and the lowest living branches in the crown of a tree) and crown width were measured in two orthogonal cardinal directions (north–south and east–west).

The vertical light profile in the forests was measured using a Photosynthetic Photon Flux Density (PPFD) sensor (DEFI2-L, JFE Advantech Co., Ltd, Hyogo, Japan) attached to a 20 m telescopic carbon rod (Taketani Trading Co., Osaka, Japan) (Onoda et al. 2014). To represent the average light environment during the wet season, all light measurements were conducted under overcast sky conditions, thus excluding the confounding effect of direct sunlight (Matsuo et al. 2022). At the center of each of the 16 subplots, PPFD was measured from 1 to 22 m height above the forest floor at height intervals of 1 m. At each height, PPFD was measured for 5 s and averaged. Relative light intensity (RLI, %) was calculated for each height in reference to PPFD above the canopy or simultaneously measured PPFD in an open area near the plot. Because the measurement of vertical light profile was labor-intensive work, we chose the 5 m × 5 m subplot to deal with a trade-off between maximizing the horizontal resolution and size of study area for a given fieldwork effort (Matsuo et al. 2022).

### Whole-tree, stem, and leaf traits in relation to LIE and LUE

To understand how species’ whole-tree, stem, and leaf traits underlie LIE and LUE, we measured these traits. For whole-tree traits, we chose tree height (H, m) as it determines access to light (Maharjan et al. 2021) and tree photosynthetic mass of a horizontal crown layer (hereafter, crown leaf mass: Mp, kg) as it determines the light interception for a give light environment (Rubio et al. 2021). For each individual, Mp was calculated based on crown area (CA, m^2^) and leaf mass per area (LMA, kg m^−2^) (Rubio et al. 2021) Eq. 1

1 $${\text{Mp }} = {\text{ LMA}} \times {\text{CA}}$$

Because larger individuals within a given species contribute more to their community biomass (species dominance), we calculated the size weighted average H and Mp for each species (i.e., species average H and Mp proportionally weighted by individual biomass within a species) for each forest stand. As a stem trait, we selected wood density (WD, g cm^−3^) because low WD is related to efficiency of vertical extension and thus light interception (Iida et al. 2012) while high WD is related to shade tolerance of the species and thus light utilization (Nock et al. 2009). For LIE, we chose leaf area (LA, m^2^) and LMA as leaf traits because they are related to the foliage distribution within the crown and thus the patterns of light interception (Norisada et al. 2021). For LUE, we chose LMA and leaf nitrogen concentration (LNC, mg g^−1^) as LMA is related to low dark respiration rate and thus an efficient use of resources while LNC determines photosynthetic capacity and thus net carbon gain (Wright et al. 2004).

Leaf traits were measured for ten individuals per species outside of the research plots. We followed standardized protocols (Cornelissen et al. 2003; Pérez-Harguindeguy et al. 2013). For leaf trait measurements, sun lit leaves of small adult trees (ca. 5 m high) were selected because of the easy access to the samples (Lohbeck et al. 2012). Leaves (excluding petiole) were photographed on a light box and leaf area was calculated using pixel counting software ImageJ. Leaves were subsequently oven-dried at 70 °C for 48 h (or until dry mass is stable) and weighed. LMA was calculated as the oven-dried mass (excluding petiole) divided by LA. To determine LNC, samples were ground to pass a 0.5 mm sieve prior analysis. Colorimetric determinations were carried out in a Bran-Luebbe AutoAnalyzer III (Norderstedt, Germany; Technicon Industrial Systems 1977) after acid digestion by the macro-Kjeldahl modified method. WD was measured based on wood cores, using an increment borer (12″ mm Suunto, Finland), or stem slices for species of which stems did not reach sufficient size (< 5 cm DBH). For each species, an average of five samples were collected (1–13 samples). The fresh volume was determined with the water displacement method. Then samples were oven-dried at 70 °C for 48 h (or until dry mass is stable) to measure the oven-dried mass. WD was calculated as oven-dried mass over fresh volume. This measurement was taken in the study area for 63 of 77 studied species (Lohbeck 2014), and data on WD for remaining species were taken from comparable studies in Mexican wet forests in Los Tuxlas (eight species) (Barajas-Morales 1987) and Las Margaritas (six species) (M. Martínez-Ramos and H. Paz, unpublished). Species’ average trait values were used although we recognize that intraspecific trait variation may play an important role in the acclimation of species adaptation along environmental gradients (Poorter and Rozendaal 2008; Nomura et al. 2023).

### Calculation of relative growth rate

Aboveground biomass of each individual was calculated based on its DBH, tree height, and species-specific wood density, using an allometric equation for tropical tree species (Chave et al. 2014) Eq. 2:

2 $${\text{M }} = \, 0.0{673} \times ({\text{D}}^{2} {\text{H}})^{0.{976}}$$

where M is aboveground biomass (kg), D is DBH (cm), H is tree height (m), and $\rho$ is wood density (g cm^−3^). For each tree, absolute biomass growth rate (AGR, kg year^−1^, Eq. 3) and relative biomass growth rate (RGR, in kg kg^−1^ year^−^.^1^, Eq. 4) were calculated as follows (Kohyama et al. 2019)

3 $${\text{AGR }} = \, \left( {{\text{M}}_{{2}0{18}} {\rm{ - }}{\text{ M}}_{{2}0{16}} } \right)/{\text{T}}$$

4 $${\text{RGR }} = \, \left( {{\text{ln M}}_{{2}0{18}} {\rm{ - }}{\text{ ln M}}_{{2}0{16}} } \right)/{\text{T}}$$

where M is the aboveground biomass of 2016 or 2018 and T is the time between the two measurements (two years).

### Calculation of light interception for each individual tree

The amount of light intercepted by each individual was calculated as the attenuation of irradiance within its crown (Onoda et al. 2014). We considered the forest as a 3D space consisting of voxels (0.125 m^3^) where each voxel size was 0.5 m width × 0.5 m width × 0.5 m height. Because light measurements were made at 1 m intervals and we considered the size of voxels of 0.5 m for each dimension, we linearly interpolated the RLI between each pair of vertical 1 m height classes, and hence calculated the RLI for the 0.5 m midpoint as an average of RLI between the two height classes. Because we measured one vertical light profile for each subplot (5 m × 5 m), we considered that the RLI for each height was identical within each subplot. We then assigned for each voxel the relative light interception (RL_voxel_) by calculating the difference between RLI at the top of the voxel (VRLI_top_) and the bottom of the voxel (VRLI_bottom_) (Eq. 5).

5 $${\text{RL}}_{{\text{voxel}}} = {\text{ VRLI}}_{{\text{top}}} {\rm{ - }}{\text{VRLI}}_{{\text{bottom}}}$$

For each tree, we identified the voxels that were located within its crown, and the amount of relative intercepted light for each tree was, therefore, calculated by summing relative light interception over these voxels.

To calculate the amount of annual light interception for each individual, the amount of relative light interception was multiplied by annual photosynthetic active radiation (PAR). The annual insolation at Loma Bonita in 2017 was 6874.5 MJ m^−2^ year^−1^ (whole year) and total insolation during the wet season in a year was 5166.4 MJ m^−2^ year^−1^ (NASA). PAR was calculated as the half of the insolation (i.e., whole year PAR = 3437.25 MJ m^−2^ year^−1^ and wet season PAR = 2583.2 MJ m^−2^ year^−1^) based on a previous study (Meek et al. 1984). To calculate the amount of light interception, we used, for evergreen species, the PAR of the whole year and for deciduous specie, that drop their leaves in the dry season, the PAR of the wet season. The amount of light interception was expressed in MJ, which allows comparison with other studies that have calculated LIE and LUE of trees (e.g., Binkley et al. 2010; Campoe et al. 2013).

When two or more individuals occupy the same voxel (overlapping crown), light interception in the voxel was equally split among the individuals. Individuals located at the edge of plots have a portion of their crown outside of the plots, where no light measurements were taken. We estimated this portion by knowing the crown area located inside the plot (CA_within_plot_/CA_total_) to adjust the amount of light intercepted by the whole crown (i.e., the light interception was doubled when half of the crown area was outside the plot). Due to the voxel size used in our study, it was difficult to evaluate the light interception of tiny individuals with crown width or lengths < 0.5 m. Therefore, these individuals were excluded from further analyses.

### Light interception efficiency and light use efficiency

To analyze how trees intercept and use light energy for their growth, RGR was analyzed as the product of LIE and LUE (Onoda et al. 2014).

6 $${\text{RGR }} = \, (M/L) \, \times \, \left( {L/M} \right) \, = {\text{ LUE }} \times {\text{ LIE}}$$

where $\Delta$ *M* (g year^−1^) is the biomass increment per tree per year, *M* (g) is the average aboveground biomass between censuses, and *L* (MJ year^−1^) is the amount of light intercepted per tree per year. $\Delta$ *M*/*L* (g MJ^−1^) is biomass gain per unit absorbed light, defined here as LUE. *L*/*M* (MJ g^−1^ year^−1^) is the amount of light interception per unit above-ground biomass per year, defined as LIE.

### Changes in species dominance

To understand how species’ growth rates determine changes in species dominance during succession, we used a three-step approach to obtain the measure of changes in species dominance. First, we calculated total biomass for each species in each forest stand and year (M_i2016_ and M_i2018_). Second, we calculated the sum of species biomass for each forest stand and year (M_total2016_ and M_total2018_). Third, we calculated the absolute biomass change of each species relative to absolute biomass change of all species in each stand over 2 years as the measure of the changes in species dominance (Eq. 7).

7 $${\text{Changes in species dominance }} = \, \left( {{\text{M}}_{{\text{i2}}0{18}} - {\text{M}}_{{\text{i2}}0{16}} } \right) \, / \, \left( {{\text{M}}_{{\text{total2}}0{18}} - {\text{M}}_{{\text{total2}}0{16}} } \right)$$

where M_i_ (kg) is a total biomass of *i*th species for a given year and forest stand, M_total_ (kg) is a total biomass of a forest stand for a given year. Therefore, changes in species dominance are determined by biomass gain of species through biomass growth of survived individuals and newly recruited individuals and biomass loss of species through tree mortality. Because initial species composition differs among different plots, we used changes in species dominance as an indicator of species replacement during succession.

### Data analysis and statistical analysis

To analyze if and to what extent growth rate of species can determine the changes in species dominance over time, we first calculated the size weighted average RGR of the species (i.e., species average RGR weighted by individual biomass within a species) as a species RGR for each forest stand. We chose RGR because RGR enables us to compare growth rate of species that differ in size (biomass), and RGR can be mathematically broken down into two underlying components (LIE and LUE) which are related to species’ light competition strategies (Onoda et al. 2014). The size weighted average value was used since larger individuals within a same species contribute more to their community biomass. To compare the effects of predictor variables with different units on RGR, we standardized the data. We standardized the RGR and changes in species dominance of each species for each stand by subtracting for each variable the mean value across species and then dividing it by the standard deviation across species for each stand (Hu et al. 2011, Eq. 8).

8 $$Z = (x - \mu )/\sigma$$

where Z represents standardized value, x represents raw value, $\mu$ represents mean, and $\sigma$ represents standard deviation. To evaluate how species RGR is related to changes in species dominance during succession, we conducted a linear mixed model using the “lme4”, “performance”, and “lmerTest” packages in R (Bates 2007; Kuznetsova et al. 2017; Lüdecke et al. 2021). The change in species dominance was included as a response variable, and species RGR and its interaction with forest age were included as fixed variables. As random variables, we included forest stands and species to account for the fact that tree species were nested within stands and to consider the species’ characteristics which may influence the patterns.

**Fig. 1:**
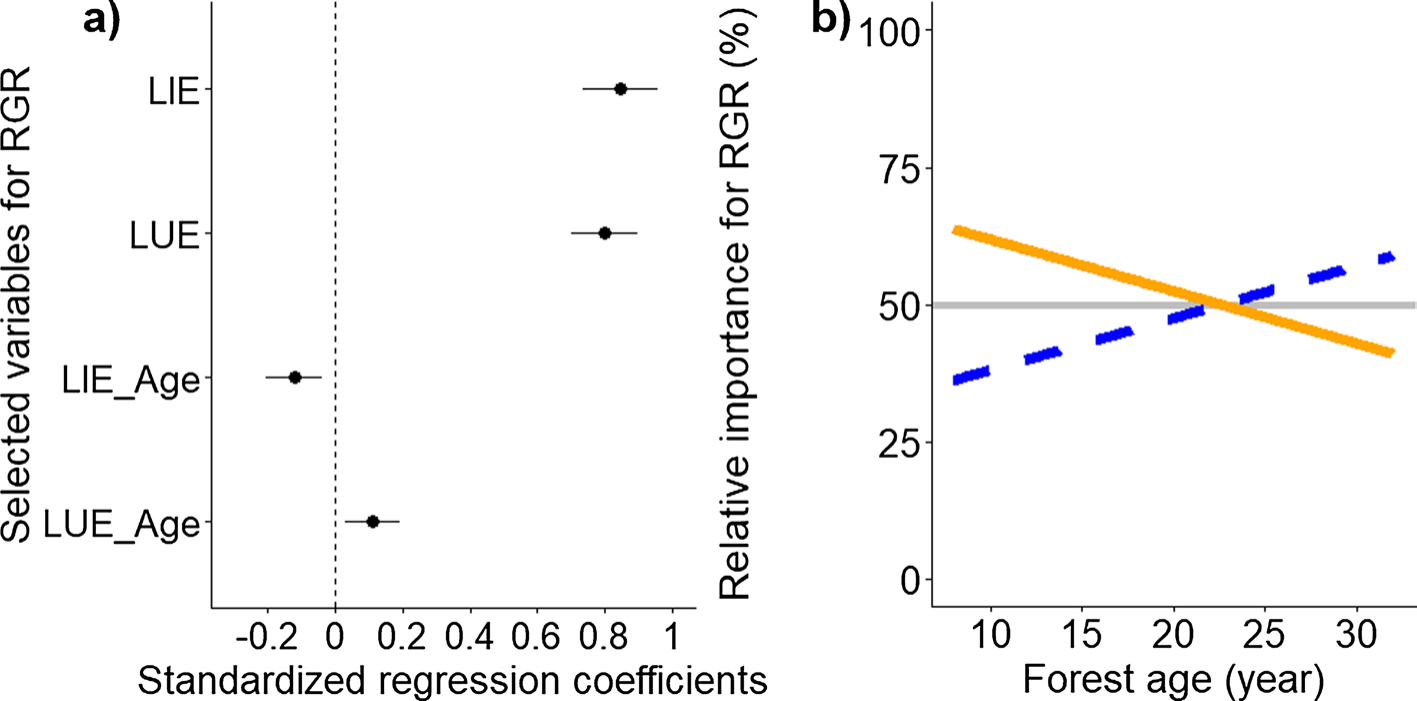
**a** The model-averaged estimates of standardized coefficients for species average relative growth rate (RGR, g g^−1^ year^−1^). The linear mixed model was conducted with species RGR as a response variable, and species light interception efficiency (LIE, MJ g^−1^ year^−1^), species light use efficiency (LUE, g MJ^−^.^1^), and their interactions with forest age (Age, year) as fixed variables. Forest stands and species were included as random factors to account for the fact that tree species were nested within stands. RGR, LIE, and LUE were log_10_-transformed prior to the standardization (Eq. 8) to improve the statistical model. The model-averaged estimates of standardized coefficients were calculated based on the best models with AICc (sample-corrected Akaike information criterion) ≤ 2 (see method for the detail). Lines represent 95% confidence intervals, while circles represent the model estimated value. Filled black circles represent significant parameters at *p* < 0.05. Refer to Tables S4, S7 for details. **b** The relative importance of LIE (continuous orange line) and LUE (dashed blue line) for RGR versus forest age based on the result of the linear mixed model (Table S7)

To understand how species RGR is determined by species LIE and species LUE during succession, we first calculated for each stand the size weighted average RGR, LIE and LUE of the species, and then standardized them (Eq. 8). We then conducted a linear mixed model with species RGR as a response variable, and species LIE, species LUE, and their interactions with forest age as fixed variables, and forest stands and species as random variables. Because RGR is the product of LIE and LUE, we divided the standardized effect size of LIE by the sum of standardized effect size of LIE and LUE multiplied with 100 for a given stand age. We used this to quantify the relative importance of LIE for RGR (%) in this study (Fig. 1a). Similarly, we calculated the relative importance of LUE for RGR for each forest stand (Fig. 1b).

To assess how functional traits determine LIE and LUE, we standardized LIE, LUE, and all traits (Eq. 8), and conducted two linear mixed models. For LIE, we conducted the model with species LIE as a response variable; and species height, Mp, LMA, LA, and WD, and their interactions with forest age as fixed variables; and forest stands and species as random variables. For LUE, we conducted the model with species LUE as a response variable; and species height, Mp, LMA, LNC, and WD, and their interactions with forest age as fixed variables; and forest stands and species as random variables.

To select the most influential variables for each model, a dredge model selection was performed using the “MuMIn” package with all possible subsets and combinations of independent variables (Barton 2012). Model selection was based on the lowest sample-corrected Akaike information criterion (AICc). Models differing ≤ 2 AICc from the best model were considered to have an equally good fit. Because the model selection with AICc using a function “dredge” chose two or three best models in our analysis (Table S3, S4, S5), we attempted model averaging (Burnham et al. 2011) to reduce model selection uncertainly. With this, we calculated the model-averaged estimates of standardized coefficients and *p* values for the averaged model using the best models by a function of “model.avg” in the “AICcmodavg” package (Tables S6–S8) (Mazerolle and Mazerolle 2017). In all models, variance inflation factors of predictor variables were lower than 3 (Tables S7, S8). In total, 77 species were included in the analyses, which accounted for 84.0% of the whole individuals of forest community. All data on changes in species dominance, RGR, LIE, LUE, and Mp were log_10_-transformed prior to the standardized transformation to improve the model by reducing the large variation among species. Basic information of forest and light attributes are shown in Fig. S1. All data were analyzed using the statistical package R (version 3.4.0; R Foundation for Statistical Computing, Vienna, Austria) (R Core Team 2013).

## Results

### How does RGR determine changes in species dominance during succession?

Throughout succession, species RGR was positively correlated with changes in species dominance (0.16, with 95% confidence interval of 0.04–0.28) (Fig. S2, Table S6), which indicates that species with higher RGR become more dominant over time. The fact that only RGR was selected in the best model indicates that RGR is consistently an important driver of changes in species dominance throughout succession (Table S6).

### Relative importance of LIE and LUE for species RGR during succession

Throughout succession, LIE and LUE both increased RGR (Table S7, Fig. 1a). Because the interaction between LIE and stand age was negative while the interaction between LUE and stand age was positive (Fig. 1a), the relative importance of LUE for RGR increased concomitantly with forest age (Fig. 1b). Hence, in early succession, species with high LIE tend to have fast RGR. After 23 years of succession, relative importance of LUE for RGR reached 50% (Fig. 1b), indicating that LIE and LUE became equally important in shaping RGR, and thereafter LUE became more important than LIE for RGR.

### How do whole-tree, stem, and leaf traits determine LIE and LUE?

**Fig. 2:**
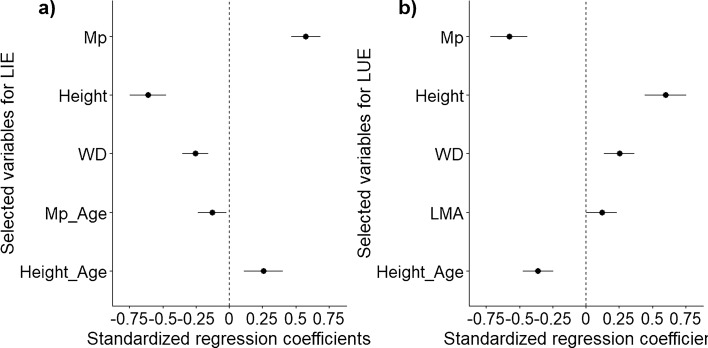
The model-averaged estimates of standardized coefficients for **a** light interception efficiency (LIE, MJ g^−1^ year^−1^) and **b** light use efficiency (LUE, g MJ^−1^). For LIE, the linear mixed model was conducted with species LIE as a response variable, and total tree photosynthetic mass of a horizontal crown layer (Mp, kg), tree height (H, m), wood density (WD, g cm^−3^), leaf mass per area (LMA, kg m^−2^), leaf area (LA, cm^2^), and their interactions with forest age (Age, year) as fixed variables. Forest stands and species were included as random factors to account for the fact that tree species were nested within stands and to consider the species’ characteristics which we did not consider through functional traits but may influence the patterns. Similarly, for LUE, we conducted the linear mixed model with species LUE as a response variable; and Mp, H, WD, LMA, leaf nitrogen concentration (LNC, mg g^−1^), and their interactions with forest age as fixed variables; and forest stands and species as random variables. LIE, LUE, and Mp were log_10_-transformed prior to the standardization (Eq. 8) to improve the statistical models. The model-averaged estimates of standardized coefficients were calculated based on the best models with AICc (sample-corrected Akaike information criterion) ≤ 2 (see Method for the detail). Lines represent 95% confidence intervals, while circles represent the model estimated value. Filled black circles represent significant parameters at *p* < 0.05. See Tables S5, S8 for details

Whole-tree traits (height and Mp) and WD were significantly correlated with species LIE and LUE during succession, while this applied to a lesser extent for the leaf traits (Table S8, Fig. 2). Mp was positively correlated with LIE and its interaction with stand age was negative, indicating that in early succession, trees with larger crown leaf mass tend to have higher LIE, and this trend declines with stand age. Height was negatively correlated with LIE and its interaction with stand age was positive (Table S8a, Fig. 2a), indicating that the negative effect of height on LIE decreases with stand age. WD was negatively correlated with LIE indicating that species with low WD consistently had higher LIE than the species with high WD throughout succession. Single leaf-level traits such as LA and LMA were not selected in the best model for LIE (Table S5a).

For LUE, Mp was negatively correlated with LUE, and its interaction with stand age was not significant (Table S8b, Fig. 2b), indicating that larger crown mass reduces LUE throughout succession. Height was positively correlated with LUE and its interaction with stand age was negative, indicating that in early succession, taller species have higher LUE and this trend declines with stand age. Species with high LMA and WD had higher LUE throughout succession, indicating that species with higher shade tolerance (i.e., high LMA and WD) are more efficient to convert light to biomass growth. LNC was not selected in the best model for LUE (Table S5b).

## Discussion

We analyzed how RGR, light competition strategies, and functional traits drive changes in species dominance during tropical forest succession. Species with higher RGR had higher changes in species dominance and thus increased their dominance over time. Both light competition strategies (LIE and LUE) increased RGR, but their relative importance shifted during succession; LIE was more important early in succession, whereas LIE and LUE became equally important for RGR after 23 years of succession. Whole-tree and stem traits were always significantly correlated with LIE and LUE while leaf traits were relatively poor predictors of LIE and LUE.

### RGR determine changes in species dominance during succession

RGR was positively correlated with the changes in species dominance throughout succession (Table S6, Fig. S2), and thus species with higher RGR increase their relative biomass in the forest stands, and become dominant over time (Muscarella et al. 2017). Although recruitment and mortality also influence successional changes in species biomass over time in both young secondary and old-growth forests (Jakovac et al. 2016; van der Sande et al. 2017), growth (i.e., RGR) was a strong determinant of species biomass and species turnover in our young secondary forest site.

Although the relationship between RGR and changes in species dominance remained similar during succession, median RGR declined during succession from 0.39 g g^−1^ year^−1^ in 8-year-old forest to 0.13 g g^−1^ year^−1^ in 32-year-old forest (Fig. S1e). Hence, the faster species growth rate in early succession comes along with a faster species turnover (Chazdon et al. 2007).

### Relative importance of LIE and LUE for RGR during succession

We found a rapid change in the relative importance of LIE and LUE for RGR during the first 20 years of succession (Table S7, Fig. 1). This successional change in the relative importance might be attributed to the successional change in forest structure and vertical light attenuation rate in this forest during the first 20 years (Matsuo et al. 2021). Early in succession, stands are more open and light availability is relatively high in all forest strata, and hence most individuals in this ‘stand initiation phase’ (Oliver 1980) are under well-lit conditions and have high leaf photosynthetic rate (Givnish 1988; Onoda et al. 2014). Therefore, larger light interception efficiency increases whole plant photosynthetic rate, and hence RGR (Van Kuijk et al. 2008). As succession proceeds, canopy height and stand AGB increase, which leads to a reduced light intensity in the lower forest strata (van Breugel et al. 2006; Matsuo et al. 2021). Hence, in the ‘stand thinning phase’ and mature forest phase, a larger numbers of trees are under light-limited conditions (Oliver 1980). Therefore, more individuals and species need to convert the limited light efficiently into carbon to attain fast growth rate, and hence LUE becomes more important for RGR over time (Kitajima et al. 2005). In sum, successional changes in forest structure and concomitant changes in forest light conditions drive a successional shift in light competition strategies from light-demanding pioneer species with efficient light interception to shade-tolerant species with an efficient light use (Selaya et al. 2007; Lasky et al. 2014).

### Whole-tree, stem, and leaf traits determine LIE and LUE during succession

Light competition strategies (LIE and LUE) were mainly determined by whole-tree and stem traits, and to a lesser extent by leaf traits (Tables S5, S8, Fig. 2).

#### Tree height

Early in succession, tree height decreased LIE whereas later in succession, tree height increased LIE. This may be related to the increasing vertical light heterogeneity in forests during succession because larger vertical light heterogeneity can drive stronger asymmetric competition for light which puts more premium for taller species for the light interception (Matsuo et al. 2021). LUE was positively related to tree height in earlier succession, but the relationship became weaker during succession (Fig. 2b). Early in succession, when pioneer species dominate, taller trees of pioneer species have higher LUE because pioneer species are often light demanding and hence can optimize their growth under full sun light whereas smaller trees of pioneer species cannot grow well under dark conditions due to their higher metabolic rates, and hence lower LUE (Binkley et al. 2010). In later succession, when shade-tolerant species become more dominant, taller canopy species have lower LUE because canopy trees cannot utilize the intercepted light efficiently due to high maintenance costs and due to the longer hydraulic pathway which induces stomatal closure during drought (Givnish 1988; Guillemot et al. 2022). In contrast, short-statured shade-tolerant species have lower metabolic rates and are able to attain positive growth rates and, hence, higher LUE under light-limited understory conditions (Valladares and Niinemets 2008; Onoda et al. 2014).

#### Tree photosynthetic mass of a horizontal crown layer

LIE was positively correlated with Mp but the correlation became weaker over time (Fig. 2a). Early in succession, light is more abundant in forests and thus larger crown leaf mass strongly determines the amount of light interception, and hence LIE (Maharjan et al. 2021; Matsuo et al. 2021). As succession advances, vertical light heterogeneity increases and tree height which determines the access to light becomes relatively more important than Mp. Mp was negatively correlated with LUE throughout succession (Fig. 2b), indicating that increasing biomass allocation to leaves may increase dark respiration rates and hence reduced LUE (Reich et al. 2008) or that leaves are relatively short-lived and have to be replaced continuously (Sterck et al. 2006a).

#### Wood density and LMA

LIE was negatively correlated with WD throughout succession because species with low WD can attain same height growth with low biomass investment, and thus can maximize light interception for a given biomass (Sterck et al. 2006b; Anten and Schieving 2010; Iida et al. 2012). In contrast, LUE was positively correlated with WD and LMA because these species tend have slow metabolic rates (Wright et al. 2004), low photosynthetic light compensation points, slow tissue turnover, and low tissue loss rates due to biophysical hazards (Kitajima et al. 2012), which increase the efficiency of intercepted light to growth (Sterck et al. 2006a; Valladares and Niinemets 2008; Nock et al. 2009).

## Conclusion

Throughout succession, species with higher RGR exhibit a faster increase in dominance over time. Both LIE and LUE contribute to RGR throughout succession, although their relative importance changes during succession. In early succession, species with high LIE and its associated traits (high crown leaf mass and low WD) attain greater RGR, and thus increase their dominance. As succession advances, forest structure builds up, leading to the lower light levels in the understory. As a result, in later succession, species with high LUE and its associated traits (high WD and LMA) attain greater RGR because of their slower metabolic rates. Therefore, successional changes in relative importance for RGR from LIE to LUE along with the concomitant shift in underlying traits from acquisitive to conservative drive the shift in species dominance from light-demanding pioneer species to shade-tolerant late-successional species during tropical rainforest succession.

### Supplementary Information

Below is the link to the electronic supplementary material.

Supplementary file1 (DOCX 417 KB)

## Data Availability

The data used for this study will be stored in DANS (Data Archiving and Networked Services) upon acceptance.
