# Supplementary material for: Light competition drives species replacement during secondary tropical forest succession
Source: Oecologia. 2024 May 10;205(1):1–11. doi: 10.1007/s00442-024-05551-w (PMC11144147; doi:10.1007/s00442-024-05551-w)
Supplement: Supplementary file 1 — Supplementary file1 (DOCX 417 KB) [file 442_2024_5551_MOESM1_ESM.docx]

**Supplementary Material**

Table S1. Species information and characteristics of 77 studied species in Loma Bonita, southeastern Mexico. The table includes family, genus and species name, and successional guild (Early-, Mid-, Late-successional species, and NA: no information), deciduousness, Dmin; minimum stem diameter at breast height (cm), Dmax; maximum stem diameter at breast height (cm), Hmin; minimum tree height (m), and Hmax; maximum tree height observed in the study site (m). The successional guild is based on the existing studies conducted in the study site and literatures in the Neotropics (M. Martínez-Ramos, unpublished).

Table S2. Top three dominant studied species based on the relative aboveground biomass (AGB) for each plot. The relative AGB for each species was calculated as the total AGB for each species divided by the total AGB for each plot, multiplied by 100. Because larger trees strongly shape the forest structure, we calculated the species dominance based on the relative AGB instead of numbers of individuals per species.


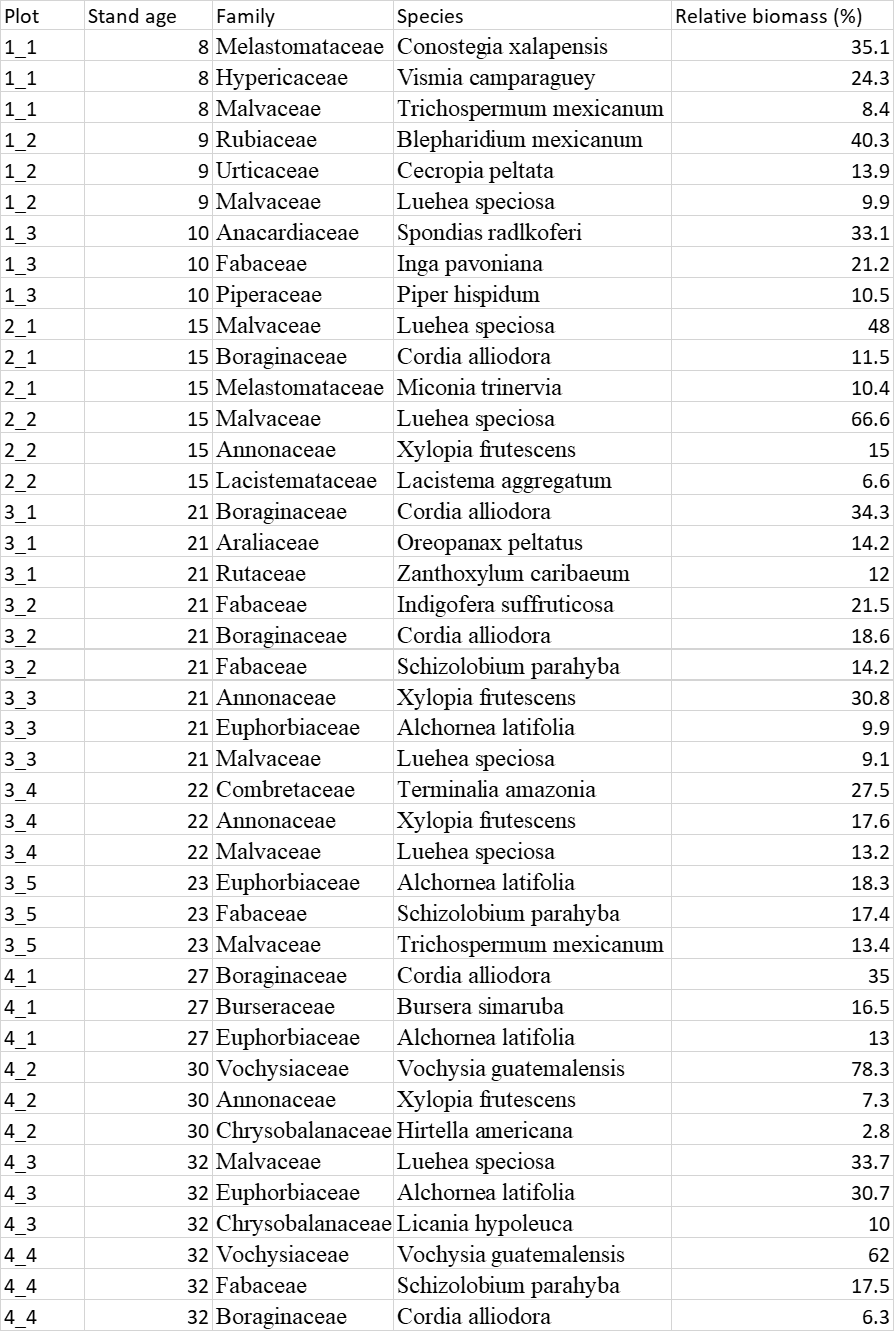


Table S3. The best models of the changes in species dominance by model selection. The table shows the set of best models with indiscernible ΔAIC ≤2 that include intercept (β_0_) and species average relative growth rate (RGR, g g^-1^ year^-1^) and its interaction with stand age (Age, year). Moreover, AICc (sample-corrected Akaike information criterion), ∆AICc (AICc_i_ − AICc_min_) and Akaike weights (*w_i_*) are shown for each model.


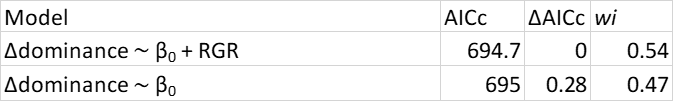


Table S4. The best models of species average relative growth rate (RGR, g g^-1^ year^-1^) by model selection. The table shows the set of best models with indiscernible ΔAICc ≤2 that include intercept (β_0_) and species average LIE (light interception efficiency, MJ g^-1^ year^-1^), species average LUE (light use efficiency, g MJ ^-1^) and their interactions with forest age (Age, year). Moreover, AICc (sample-corrected Akaike information criterion), ∆AICc (AICc_i_ − AICc_min_) and Akaike weights (*w_i_*) are shown for each model.


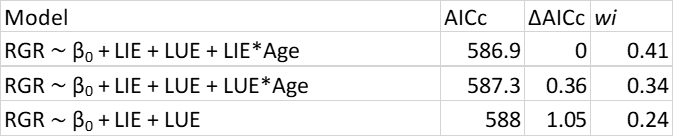


Table S5. The best models of a) species average LIE (light interception efficiency, MJ g^-1^ year^-1^) and b) species average LUE (light use efficiency, g MJ ^-1^) by modl selection. The table shows the set of best models with indiscernible ΔAICc ≤ 2 that include intercept (β_0_) and traits (refer to Table E8 for the abbreviations) and their interactions with forest age (Age, year). Moreover, AICc (sample-corrected Akaike information criterion), ∆AICc (AICc_i_ − AICc_min_) and Akaike weights (*w_i_*) are shown for each model.


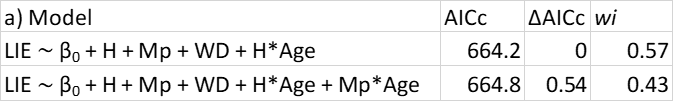


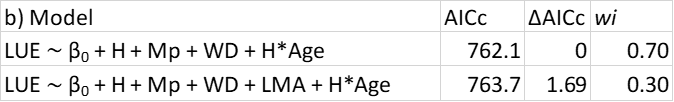


Table S6. Summary of the model-averaged estimates of standardized coefficients for the changes in species dominance. The linear mixed model was conducted with changes in species dominance as a response variable, and species average relative growth rate (g g^-1^ year^-1^) and its interaction with forest age (Age, year) as fixed variables. As random variables, we included forest stands and species to account for the fact that tree species were nested within stands. Changes in species dominance and relative growth rate were log_10_-transformed prior to the standardization (eq. 8) to improve the model. The most influential variables were selected based on the best models with AICc (sample-corrected Akaike information criterion) ≤ 2 (see method for the detail). Because the model selection with AICc using a function “dredge” chose two or three best models in our analysis (Table S3), we attempted model averaging to reduce model selection uncertainly. With this, we calculated the model-averaged estimates of standardized coefficients and p-values for the averaged model using the best models by a function of “model.avg” in the “AICcmodavg” package.


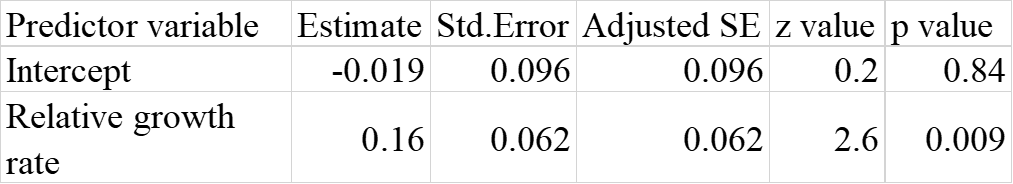


Table S7. Summary of the model-averaged estimates of standardized coefficients for species average relative growth rate (RGR, g g^-1^ year^-1^). The linear mixed model was conducted with species RGR as a response variable, and species average LIE (light interception efficiency, MJ g^-1^ year^-1^), LUE (light use efficiency, g MJ ^-1^) and their interactions with forest age (Age, year) as fixed variables. As random variables, we included forest stands and species to account for the fact that tree species were nested within stands. RGR, LIE and LUE were log_10_-transformed prior to the standardization (eq. 8) to improve the model. The most influential variables for each model were selected based on the best models with AICc (sample-corrected Akaike information criterion) ≤ 2 (see method for the detail). Variance inflation factor (VIF) is shown for each predictor variable. Because the model selection with AICc using a function “dredge” chose two or three best models in our analysis (Table S4), we attempted model averaging to reduce model selection uncertainly. With this, we calculated the model-averaged estimates of standardized coefficients and p-values for the averaged model using the best models by a function of “model.avg” in the “AICcmodavg” package.

Table S8. Summary of the model-averaged estimates of standardized coefficients for the effects of traits on a) light interception efficiency (LIE, MJ g^-1^ year^-1^) and b) light use efficiency (LUE, g MJ ^-1^). The linear mixed model was conducted with species average LIE as a response variable, and total tree photosynthetic mass of a horizontal crown layer (Mp, kg), tree height (H, m), wood density (WD, g cm^-3^), leaf mass per area (LMA, kg m^-2^), leaf area (LA, cm^2^) and their interactions with forest age as fixed variables. Forest stands and species were included as random variable to account for the fact that tree species were nested within stands and to consider the species’ characteristics which we did not consider through functional traits but may influence the patterns. Similarly, we conducted the linear mixed model with species LUE as a response variable, and Mp, H, WD, LMA, leaf nitrogen concentration (LNC, mg g^-1^) and their interactions with forest age as fixed variables, and forest stands and species as random variables. LIE, LUE and Mp were log_10_-transformed prior to the standardization (eq. 8) to improve the statistical models. The most influential variables for each model were selected based on the best models with AICc (sample-corrected Akaike information criterion) ≤ 2 (see method for the detail). Variance inflation factor (VIF) is shown for each predictor variable. Because the model selection with AICc using a function “dredge” chose two or three best models in our analysis (Table S5), we attempted model averaging to reduce model selection uncertainly. With this, we calculated the model-averaged estimates of standardized coefficients and p-values for the averaged model using the best models by a function of “model.avg” in the “AICcmodavg” package.


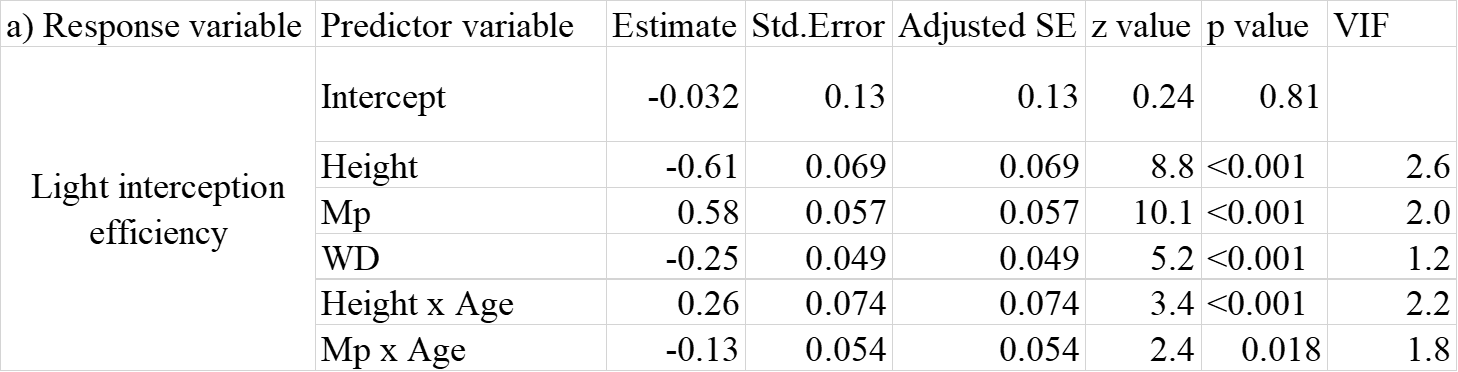

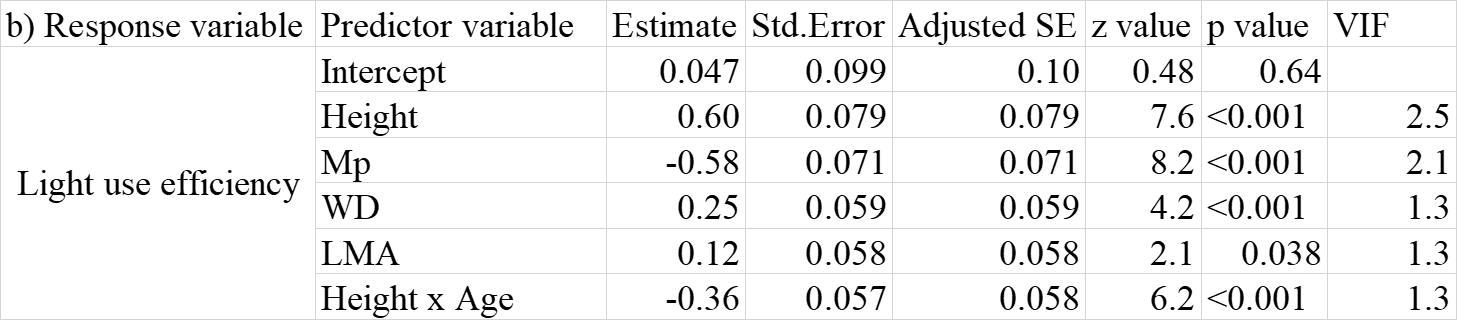


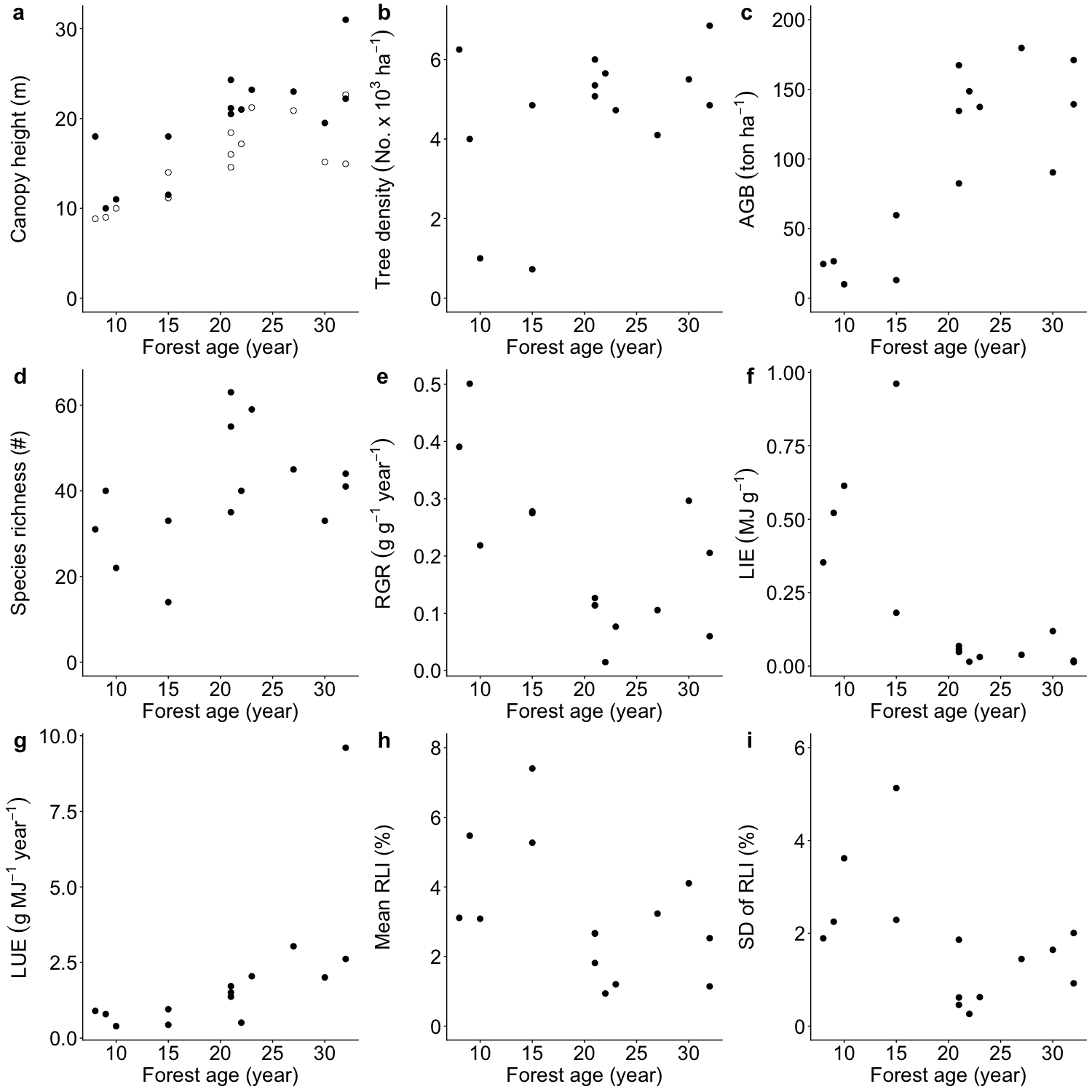


Figure S1. Forest and light attributes during succession. a) canopy height (the heights of tallest tree: closed circles and the height of the top 5% tallest trees: open circles), b) numbers of tree individuals (Tree density), (c) total aboveground biomass (AGB), d) numbers of species (species richness), median values of e) RGR (relative growth rate), f) LIE (light interception efficiency), and g) LUE (light use efficiency), and mean and standard deviation (SD) of relative light intensity (RLI, %) at 1 m from the ground versus forest age. Due to the several gap formations and associated frequent sunflecks in one of the 32-year-old forests, median value of LUE was relatively high.


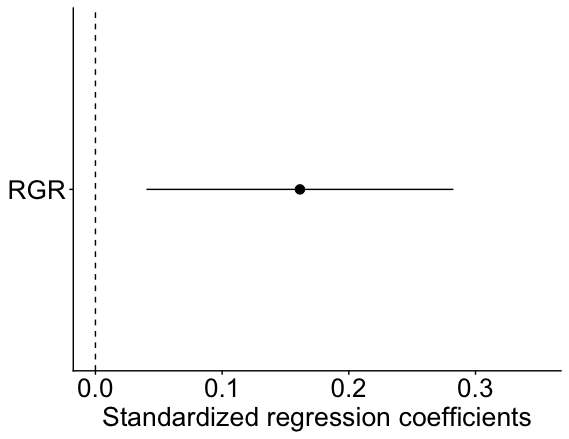


Figure S2. Standardized regression coefficient for the changes in species dominance. The linear mixed model was conducted with changes in species dominance as a response variable, and species average RGR (relative growth rate, g g^-1^ year^-1^) and its interaction with forest age as fixed variables. As random variables, we included forest stands and species to account for the fact that tree species were nested within stands. Changes in species dominance and RGR were log_10_-transformed prior to the standardization (eq. 8) to improve the model. The most influential variables for each model were selected based on the best models with AICc (Akaike information criterion) ≤ 2 (see method for the detail). Lines represent 95% confidence intervals, while circles represent the model estimated value. A filled black circle represents a significant parameter at P < 0.05. Refer to Table S3, S4 for details.
